# Regulation of blood flow and volume exchange across the microcirculation

**DOI:** 10.1186/s13054-016-1485-0

**Published:** 2016-10-21

**Authors:** Matthias Jacob, Daniel Chappell, Bernhard F. Becker

**Affiliations:** 1 Department of Anaesthesiology, St. Elisabeth Hospital Straubing, St. Elisabeth Str. 23, 94315 Straubing, Germany; 2 Department of Anaesthesiology, University Hospital Munich, Munich, Germany; 3 Walter Brendel Centre of Experimental Medicine, Ludwig-Maximilians-University Munich, St.-Elisabeth-Str., Munich, Germany

**Keywords:** Microcirculation, Endothelium, Glycocalyx, Blood flow, Tissue oxygenation, Blood vessels

## Abstract

Oxygen delivery to cells is the basic prerequisite of life. Within the human body, an ingenious oxygen delivery system, comprising steps of convection and diffusion from the upper airways via the lungs and the cardiovascular system to the microvascular area, bridges the gap between oxygen in the outside airspace and the interstitial space around the cells. However, the complexity of this evolutionary development makes us prone to pathophysiological problems. While those problems related to respiration and macrohemodynamics have already been successfully addressed by modern medicine, the pathophysiology of the microcirculation is still often a closed book in daily practice. Nevertheless, here as well, profound physiological understanding is the only key to rational therapeutic decisions. The prime guarantor of tissue oxygenation is tissue blood flow. Therefore, on the premise of intact macrohemodynamics, the microcirculation has three major responsibilities: 1) providing access for oxygenated blood to the tissues and appropriate return of volume; 2) maintaining global tissue flood flow, even in the face of changes in central blood pressure; and 3) linking local blood flow to local metabolic needs. It is an intriguing concept of nature to do this mainly by local regulatory mechanisms, impacting primarily on flow resistance, be this via endothelial or direct smooth muscle actions. The final goal of microvascular blood flow per unit of time is to ensure the needed exchange of substances between tissue and blood compartments. The two principle means of accomplishing this are diffusion and filtration. While simple diffusion is the quantitatively most important form of capillary exchange activity for the respiratory gases, water flux across the blood-brain barrier is facilitated via preformed specialized channels, the aquaporines. Beyond that, the vascular barrier is practically nowhere completely tight for water, with paracellular filtration giving rise to generally low but permanent fluid flux outwards into the interstitial space at the microvascular high pressure segment. At the more leaky venular aspect, both filtration and diffusion allow for bidirectional passage of water, nutrients, and waste products. We are just beginning to appreciate that a major factor for maintaining tissue fluid homeostasis appears to be the integrity of the endothelial glycocalyx.

## Background

Single cell eucaryotes communicate directly with their aqueous environment to extract oxygen (O_2_) and nutrients, and to excrete carbon dioxide (CO_2_) and waste products, via the cell surface. This facile exchange modality is not available to cells of higher organisms since these have organ systems and tissue compartments with a relatively tight outer layer which hardly lets water pass through. The formerly huge aqueous environment is now small and inside the body and is named interstitial space. All in all, this space comprises around 15 liters in the cardiopulmonarily healthy male adult. A small part of the extracellular compartment, termed “plasma”, takes part in a new evolutionary development called “circulation”. This bridges the gap between the O_2_ and nutrients available from within the lungs or the digestive tract and the interstitial space around the cells with the help of the external work performed by a newly acquired organ named the “heart”. To fulfill its pump-like function, the heart is connected to the complex system of further organs and body parts via conduits, the self-contained vascular system. This is essential, because one truth holds also for cells within complex organ systems: oxygenation at the cellular level is the basic prerequisite for maintaining aerobic metabolism, enabling the maximal possible gain of adenosine triphosphate (ATP) per molecule of glucose while avoiding large-scale production of lactic acid (see Eqs. 1 and 2).

Equation 1: net energy output of metabolizing 1 molecule of blood glucose via aerobic glycolysis

$$ 1\ \mathrm{Glucose} + 6\ {\mathrm{O}}_2 + 38\ \mathrm{A}\mathrm{D}\mathrm{P} + 38\ \mathrm{P}\ \to\ 6\ \mathrm{C}{\mathrm{O}}_2 + 44\ {\mathrm{H}}_2\mathrm{O} + \underline {\mathbf{38}\ \mathbf{A}\mathbf{T}\mathbf{P}} $$

where ADP = adenosine diphosphate, CO_2_ = carbon dioxide; P = phosphate, H_2_O = water. This equation holds true allowing that the 2 molecules of GTP initially formed in the citrate cycle per molecule of glucose are converted to ATP (see Ganong [1]).

Equation 2: net energy output of metabolizing 1 molecule of blood glucose via anaerobic glycolysis

$$ 1\ \mathrm{Glucose} + 4\ \mathrm{NADH} + 4\ {\mathrm{H}}^{+} + 2\ \mathrm{A}\mathrm{D}\mathrm{P} + 2\ \mathrm{P}\ \to\ 2\ {\mathrm{Lactate}}^{\hbox{-} } + 2\ {\mathrm{H}}^{+} + 4\ {\mathrm{NAD}}^{+}+\underline {\mathbf{2}\ \mathbf{A}\mathbf{T}\mathbf{P}} $$

where NAD^+^/ NADH = nicotinamide adenine dinucleotide oxidized/reduced, H^+^ = proton.

While the waste products of aerobic glycolysis are normally not too much of a problem, accumulation of lactic acid under anaerobic conditions leads to metabolic acidosis, accentuating negative side effects as encountered in hypoxic tissue. These include loss of cardiac contractility, loss of circulatory resistance regulation, a delayed and disturbed tissue healing process in the traumatized and critically ill patient, and, ultimately, death [2, 3]. Poorer cardiac contractility and loss of peripheral arterial resistance generally develop in acidotic settings, be these of respiratory or metabolic genesis. The decisive issue is the perturbation of the H^+^ ion concentration. In particular, increase of H^+^ attenuates glycolytic enzymes and causes hyperkalemia. These effects alone lead to a fall in cardiac force development. However, there may be some difference between metabolic and respiratory acidosis. The difference is not one of a general nature, but is quantitative. For instance, nonrespiratory acidosis causes an increase in external K^+^ of 0.6 mmol/l per change of pH by 0.1 unit; the corresponding value for respiratory acidosis is only 0.1 mmol/l per 0.1 pH unit. Also, quantitative effects will probably differ between acute and chronic acidosis. Lactate is chiefly a marker of the condition, but, as an osmotically active particle, interstitial lactate adds to vascular dilatation via transient receptor potential-operated channels of the smooth muscle cells that respond to osmotic concentration (see below).

Obviously, anaerobic glycolysis is not really a suitable alternative to the aerobic version of generating metabolic energy. Unfortunately, the latter requires oxygen.

### Oxygen delivery—from simple diffusion to a complex cascade

Over the course of a lifespan, an average human being will consume about 12 million liters of O_2_. The problem associated with trying to conduct this mass transport alone via diffusion is the overproportional increase in time required for diffusion over growing distances, as deduced from the second law of diffusion first derived by Fick [4]. While only about 2 μs are required for a molecule of oxygen to diffuse across 0.1 μm, the closest separation between a red blood cell and a capillary endothelial cell, 0.5 ms are required in order to transport O_2_ over 1 μm, i.e., the width of an endothelial cell. Though this is still readily compatible with physiological metabolic rates, for O_2_ to diffuse a distance of 1 cm would take 15 h, a totally inacceptable situation. A human cardiovascular system, however, takes oxygen from the lung to any point in the body within 30–60 s. Therefore, all larger organisms need a circulatory system that provides efficient convective transport in addition to diffusion. Nevertheless, considering Fick’s first law of diffusion, it would still be advantageous for larger bodies to establish close contact between convective and diffusional transport sequences, and nature has done just that in designing a circulatory system composed of macro- and microcirculatory segments [5]. Accordingly, the formerly relatively simple supply of single cells with O_2_ is now, within the higher organism, closely related to an intact cascade of: i) convection from outside through the airways towards the lungs to generate an alveolar partial pressure of oxygen (pO_2_) of around 100 mmHg at ambient air pressure (Eq. 3); ii) diffusion along a pO_2_ gradient from the inner alveolar surface to the lung microvessels filled with blood coming back from the periphery with a pO_2_ of around 40 mmHg (distance from gas to blood phase about 0.7 μm); iii) pulsatile convection with the central blood stream towards the evenly perfused parts of the microcirculation, with cardiac output (CO) together with the arterial oxygen content (CaO_2_) defining global oxygen delivery (DO_2_; Eq. 4); and, finally, iv) diffusion towards the cells (and ultimately to their mitochondria) along a concentration gradient.

Equation 3: the alveolar gas equation

$$ \mathrm{p}\mathrm{A}{\mathrm{O}}_2 = \left(\left({\mathrm{p}}_{\mathrm{amb}} - \mathrm{p}{\mathrm{H}}_2{\mathrm{O}}_{\mathrm{sat}}\right) \times \mathrm{F}\mathrm{i}{\mathrm{O}}_2\right)\ \hbox{--}\ \left(\mathrm{p}\mathrm{a}\mathrm{C}{\mathrm{O}}_2/\mathrm{R}\mathrm{Q}\right) $$

where pAO_2_ = alveolar partial pressure of oxygen, p_amb_ = ambient air pressure, pH_2_O_sat_ = saturated water vapor pressure, paCO_2_ = arterial partial pressure of carbon dioxide, FiO_2_ = fractional oxygen content of inspiratory gas, RQ = respiratory quotient.

Thus, under normal room air steady-state conditions, pAO_2_ = ((760 mmHg – 47 mmHg) × 0.21) – (40 mmHg / 0.8) = 100 mmHg

Equation 4: The determinants of oxygen delivery

$$ \mathrm{D}{\mathrm{O}}_2 = \mathrm{C}\mathrm{a}{\mathrm{O}}_2 \times \mathrm{C}\mathrm{O} = \mathrm{S}\mathrm{a}{\mathrm{O}}_2 \times \mathrm{c}\mathrm{H}\mathrm{b} \times 1.39 \times \mathrm{C}\mathrm{O} $$

where SaO_2_ = fractional arterial oxygen saturation of hemoglobin, cHb = hemoglobin concentration of the blood, 1.39 = Hüfner’s number (calculated).

Thus, under exemplary steady state conditions in a male adult at rest, DO_2_ = 1.0 × 14.5 gHb/dL blood × 1.39 ml O_2_/gHb × 50 dl/min = 1000 ml O_2_/min

It is remarkable that, under steady-state conditions in the human body, globally only around 25 % of the delivered oxygen is extracted per unit of time, albeit with high local differences. However, a central venous oxygen saturation of below 70 % is a well-established clinical sign that the organism might currently be getting into difficulties.

The products of aerobic glycolysis are CO_2_ and water (Eq. 1). CO_2_ has to be taken back to the lungs, again with steps of diffusion and convection (plus catalyzed conversion to carbonic acid and dissociation to bicarbonate as intermediates), while the transport of water involves some additional shunting via the lymphatic system and targets other organs besides the lungs. The transport cascade for nutrients and waste products is a comparable one, with the gut, the liver, and the kidneys replacing the lungs. In the following, we will restrict discussion to oxygenation to keep matters as simple as possible, because two things are crystal clear: 1) cells and tissues need O_2_ first of all in order to survive; and 2) O_2_ can be delivered effectively only by blood flow at the microcirculatory level [6].

Making this complex transport cascade work requires: i) open airways and intact respiratory mechanics; ii) normal dimensions and properties of the air-blood-barrier in the lung; iii) adequate hemoglobin levels and intact macrohemodynamics; and, finally and importantly, iv) an adequate distribution of microcirculatory blood flow to supply all organs with adequate amounts of O_2_ at high pO_2_ levels for those parenchymal cells ready to take up and use the O_2_. It is important to grasp that, in the fourth part of the cascade, a high partial pressure of intravascular O_2_ is needed to drive its diffusional mass transport to the mitochondria (Fick’s first law of diffusion), thus ensuring aerobic function [6].

### Critical illness—a threat to all levels of the oxygen delivery cascade

In the critically ill, the DO_2_ cascade can be endangered at all levels by various problems, e.g., by airway obstruction, pneumonia, low hemoglobin levels, circulatory collapse and/or dysregulation of the local blood flow, as in sepsis, or by difficulty in the cells taking up and using the principally available O_2_ [7]. The terms hypoxic hypoxia, ischemic hypoxia, anemic hypoxia, and toxic hypoxia serve to define situations of inadequate oxygenation as listed above [8]. Curiously, in the clinical routine of the operating theater and the intensive care unit (ICU) we are forceful in caring for: 1) blood oxygenation, e.g., by optimizing the alveolar ventilation and, when in doubt, supplementing it by extracorporeal techniques; 2) blood composition, e.g., by transfusing red cells if clinical transfusion triggers or Eq. 4 indicate this might be a good idea; and 3) adequate macrohemodynamics, foremost by use of volume, inotropes, catecholamines, and related drugs.

With respect to macrohemodynamics especially, we have not only markedly improved our treatment options but also our monitoring capabilities over the past years. The latter involves clinicians increasingly moving from monitoring of cardiac filling pressures towards flow-related parameters representing CO [9]. We have reason to be extremely happy about this success; however, this shift does not help to solve our major problem: our view of the patient still usually ends here, at the macrohemodynamic level. Thus, we are still blind for the quality of actual tissue oxygenation, i.e., the end of the DO_2_ cascade [7]. Promising bedside techniques representing local tissue perfusion such as, for example, the Sidestream Dark Field Imaging (SDF) technique are still experimental and currently restricted to only a few peripheral tissues such as the sublingual microcirculation [10, 11]. Beyond that, it is still difficult to determine an adequate reference tissue or to define normal values allowing a reproducible distinction between “normal” and “abnormal”. Therefore, we normally do not know anything about the local distribution of perfusion or of cellular uptake of the provided oxygen by the tissues of interest. Present day monitoring, as with pulse oximetry, ends up with measures representing global DO_2_ and still actively ignores the home stretch of DO_2_: that to the cells. This might be enough to appease the conscience of clinicians in 2016, but it definitely seems insufficient from a scientific point of view.

This present work looks below the surface, with the aim of keeping clinicians’ minds open to the real problems of our patients, despite the fact that our monitoring and treatment options are still limited in this regard.

### Microvascular perfusion and oxygen uptake—what do we know?

What happens when vital organs are cut off partly or completely from oxygen can be observed in the context of the development of shock organs [12]. As deducible from Eq. 4, this might be related to impaired macrohemodynamics, e.g., due to massive bleeding in trauma, acute heart failure, or excessive vasodilatation due to anaphylaxis or inflammation. Such conditions can easily be identified by the routine monitoring we apply nowadays to critically ill patients. However, in septic patients we occasionally observe the development of shock organs despite seemingly intact macrohemodynamics, indicating a problem with circulatory collapse and/or dysregulation of the local blood flow as described above, for which we are still blind. Obviously, mitochondrial dysfunction, a deficit in using the globally provided oxygen, can diminish ATP production despite high intracellular pO_2_ levels, a condition termed cytopathic or toxic hypoxia [13]. Beyond that, maldistributive shock resulting from a problem with directing the blood flow towards the tissues with high metabolic levels might also contribute to the clinically well known oxygen extraction deficit [14]. A case to point out is the condition known as vascular steal. In the myocardium especially, excessive additional dilatation of “healthy” arteriolar vessels can drain blood supply from atherosclerotic coronary vessels, where near maximal poststenotic dilatation was hitherto keeping oxygen supply to the dependent myocardial tissue at a satisfactory level. Therefore, it is possible that tissues might suffer from severe hypoxia, despite an absolutely adequate level of global DO_2_. In line with this, it has recently been found that the venous-to-arterial difference in the partial pressure of carbon dioxide (p_v-a_CO_2_) reflects microcirculatory alterations in patients with septic shock, even when global venous O_2_ saturation and cardiac output look normal [15].

Principally, in order to maintain tissue oxygenation, the microcirculation has to handle two major problems: 1) maintain global blood flow, i.e., DO_2_, to the tissues even in the face of a drop in central blood pressure (within an acceptable range); and 2) direct an adequate blood flow to tissue regions with higher metabolic needs [7]. This has been addressed by defining two distinct functional aspects of the microcirculatory section, these being “resistance” and “exchange” [16]. It appears reasonable that, based on adequate macrohemodynamics, some kind of local (auto)regulation should care for this local fine tuning. Ample experimental work from around the 1970s addressed the question of the underlying models and mechanisms [17].

There are functionally differentiated sections of the vascular bed to coordinate net external heart work with intravascular blood pressure, with cardiac preload and afterload being important parameters. This coordination serves to maintain suitable levels of DO_2_ for all the different organs [16]. Beyond an adaptation of total body flow resistance to maintain blood flow over a wide range of blood pressures, there is obviously also a variability of flow distribution to variable numbers and regions of exchange vessels, so as to maintain tissue pO_2_ above a critical level even in the face of local metabolic stress. The mechanism developed to achieve this goal is the coupling of vascular smooth muscle tone to metabolic activity of the subserved parenchym.

It is crucial to understand that regional tissue pO_2_ is a function of regional tissue blood flow and flow distribution. Regional tissue blood pressure in feed arteries is only instrumental for achieving an adequate regional tissue blood flow, reacting to regional microvascular flow resistance which, in turn, is generally regulated by vascular smooth muscle tone. The lower the regional resistance, the lower the regional blood pressure required to reach the same regional blood flow as before microvascular relaxation, i.e., to attain the same level of oxygenation at a given metabolic rate and, thus, to ensure tissue oxygenation [18].

Nevertheless, totally relinquishing microvascular resistance is not a physiologically viable option, because this would mean foregoing regulatory flexibility. In addition, maximal dilatation of all peripheral vessels would place an enormous demand of about 50 l/min CO on the pumping capacity of the heart. Therefore, an intermediate level of vascular smooth muscle tone is generally established, allowing for regulatory responses in either direction: vascular constriction or relaxation. Also, organs differ in their perfusion pressure demands. For example, working skeletal, cardiac, and gastro-intestinal muscles all need a high pressure head to ensure perfusion. This requires a cardiac pump principally able to satisfy the global demand and a vascular system able to direct the blood flow according to the local tissue needs.

### The anatomical view

The organ vasculature starting from the main organ artery connected to the aorta has been anatomically and functionally subclassified into 1) large and medium-sized “Windkessel” arteries, 2) smaller feed arteries and terminal arterioles, the latter with many collaterals and also known as the precapillary resistance arterioles, 3) exchange vessels representing the capillaries in the strict sense of the word (the “true” capillaries without any contractile elements), which drain into 4) postcapillary resistance venules and collecting veins, and finally into 5) more voluminous venous capacitance vessels and large veins. The presence of significant flow resistance in segment 4 is reflected by the fact that intravascular pressure falls by about 10–12 mmHg from the endcapillary to the larger venous segment, and then only by another 3–5 mmHg on to the heart. The existence of precapillary sphincter vessels, also termed metarterioles, described in early reviews [16], never made it beyond the frog. Segments 1 and 2 are traditionally classified as the high-pressure system; regions 4 and 5 are usually counted as belonging to the low-pressure system. Depending on posture, region 3 may belong to either the low- or the high-pressure system. It bears mentioning that walls of veins are generally less stiff than those of arteries. This is due to both cellular and fibrous composition and lower smooth muscle tone. In fact, the low-pressure system exhibits a coefficient of volume elasticity that is only about 1/25th that of the high-pressure system. In other words, if adding 1 ml of volume to the arterial system would raise pressure by 1 mmHg, then 25 ml need to be added to the venous compartment to raise pressure by the same amount. Regrettably, it is not possible to selectively load just the arterial compartment; 24 parts of 25 parts of extra volume are simply pressed out.

Interestingly, the total length of venules and collecting veins in the human body is estimated to be about 20,000 km and that of the larger veins about 450 km; large arteries amount to less than 5 meters in total length. The 20,000 km of the smallest to small venular vessels indeed harbor a large part of the total blood volume of around 5 liters. Since they exhibit an average diameter of only about 10–15 μm, this volume can be calculated to amount to about 1.5–2.5 liters. Under normal conditions, the 450 km of larger collecting and capacitance veins contain about 1–2 liters of blood (estimated average diameter 50–80 μm). Textbooks assign 80–85 % of the total blood volume to the low-pressure system. Thus, the remaining blood to be found in the arterial segments (0.7–1 liter) amounts to about 15–20 % of the total blood volume. However, the distinction between the smallest and larger low-pressure venular and venous vasculature is misleading when it comes to the question of volume recruitment and volume deposition. Irrespective of the location in the low -pressure bed, this blood can be centralized by activation of the physiological volume regulatory mechanisms (sympathetic nervous tone, vasopressin, natriuretic hormones, vascular permeability, and lymphatic return) and this is where the body initially deposits 80–85 % of any infused volume.

**Fig. 1:**
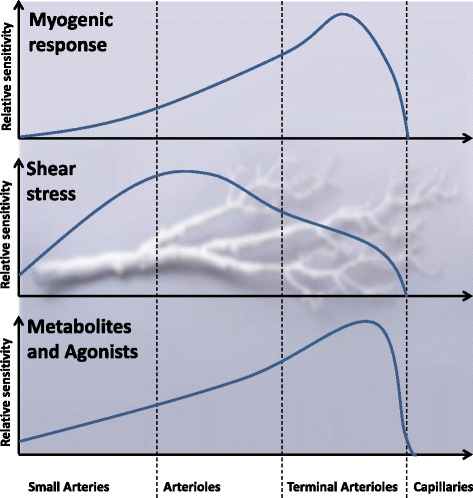
Autoregulatory responsiveness of smooth muscle cells to stimuli within the different vessel segments. The reaction of resistance to signals differs between different segments of the increasingly branching vascular tree. It is reasonable that metabolic impact can be found mainly close to the capillary diffusion and exchange area while the influence of hemodynamics is more prominent within the sections next to the large arteries (further explanations in the text)

It is worth mentioning at this point that the vascular smooth muscle cells (VSMC) of the different vessel segments differ in their electrical coupling and in their responsiveness to stimuli, partly explaining different zones of influence of diverse dilator and constrictor mechanisms (Fig. 1). VSMC of the “multi-unit” type are found in arteries, the outer layer of arterioles and the veins, giving rise to single cell responses. VSMC of the arteriolar inner layer are syncytial and behave as “single units”. The former are strongly influenced by the autonomic nervous system, while the latter respond more to local metabolic and circulating stimuli, which will be discussed in greater detail below. Of course, there are gradual transitions in response characteristic between these two extreme types of VSMC behavior [19].

It has to be understood that we are talking about an increasingly branching and reconverging system of conduits, always with the same serial anatomical principle, with two main exceptions: the glomerular capillaries and regions where arteriovenous shunts exclude the capillary exchange region. Especially within those parts of the body contributing to temperature regulation (e.g., skin of the extremities, such as fingers, ears, toes, etc.) there are true arteriovenous (AV) shunts. However, the blood stream from the arterioles to the venules also in other tissues and regions thereof may pass through a variable number of capillaries taking part in microcirculation. This phenomenon, regulated by arteriolar resistance and known as capillary recruitment, allows for modulation of the exchange area between blood and parenchymal cells. It is particularly prevalent in skeletal and heart muscle and in the lungs. Perfused capillary density may increase from 1000 to 4000/mm^2^ in the myocardium during maximal work load [20]; in the case of the lungs, recruitment is more a question of the microvessels in normally underperfused (apical) segments participating in flow at higher CO.

With increasing distance from the arteriolar side, venules begin to acquire adventitial smooth muscle cells and innervation by the autonomic adrenergic nervous system. It is still largely unclear what regulates their tone, but altered diameter will influence the blood pressure “upstream” in the capillary segments. Inflammatory growth factors such as VEGF have been reported to act as venodilators [21]. On the other hand, in inflammatory and in postischemic, reperfused tissue, leukocytes adhere in large numbers to the walls of the venules and small collecting veins [22]. This will cause partial obstruction to flow, also increasing upstream intravascular pressure with consequences for fluid filtration, a topic to be discussed more fully below. Also, inflammatory conditions can lead to architectural rearrangements of microvascular beds, including elongation of venules [23]. If nothing else, elongation of venules will prolong red blood cell residence time within the tissue, thus enabling greater exchange of respiratory gases. Low venous hemoglobin saturation need, therefore, not necessarily reflect poor oxygenation of an organ, but, rather, enhanced oxygen extraction, i.e., a beneficial adaptation.

### The physiological view

The pulsatile central blood flow with its velocity of around 20 cm/s in the aorta at a cross-sectional area of around 4 cm^2^ is dampened into a more even flow typical for the periphery by the Windkessel arteries and the precapillary resistance vessels. The total peripheral cross-sectional area in the exchange vessels, i.e., capillaries and venules, is estimated to be between 2000 and 3000 cm^2^, allowing for a mean velocity of blood flow in the order of now only 0.5 mm/s. The microvessels of the lungs provide an even larger cross-sectional area of about 4000 cm^2^. Such slow rates of passage clearly will benefit exchange processes between blood and parenchyma.

Starting at diameters of just under 300 μm, small arteries begin contributing to peripheral resistance. However, arterioles and terminal arterioles maintain and regulate the tissue blood flow by generating the major part of regional flow resistance. Table 1 lists the principle categories of physical and biological signals able to act at these vascular sites, and Fig. 1 shows that there are different sites of predilection for them to act in the arteriolar segments. In venules and small collecting veins, on the other hand, there seems to be little regulatory ability, and resistance to flow chiefly obeys the law of Hagen-Poiseuille, i.e., it is inversely proportional to the radius to the power of four and directly proportional to the length. The large number of venules (parallel resistances) initially offsets much of their contribution to global peripheral flow resistance. This contribution increases with increasing convergence. Notwithstanding, postcapillary resistance posed by venular vessels and veins is functionally important because their flow resistance contributes to determining the hydrostatic pressure upstream within the microvascular exchange section and, therefore, to the driving force of transvascular fluid filtration.

**Table 1:** Regulation of organ perfusion—the principle categories of physical and biological signals able to act at arterioles and terminal arterioles

• Myogenic reaction (Bayliss Reflex) • Neurovegetative control • Humoral- and tissue-generated mediators • Local metabolic regulation • Autacoids and shear stress (nitric oxide)	

Besides supply, one must consider exchange in the microcirculatory bed. As already mentioned, the size of the regional exchange area can be determined by the number of perfused capillaries [24]. They can be viewed as the target of all perfusion struggles: the more of them that are perfused the greater the exchange of the blood with the tissues and the smaller the diffusion distance between capillaries and cells. It appears sensible that high metabolic activity, as well as tissue hypoxia, should be connected to an (auto)regulatory dilatation of the local arteriolar resistance vessels to increase the local microcirculatory exchange area [25].

The smooth muscle tone of the capacitance vessels finally contributes to the stressed volume of the cardiovascular system and, therefore, helps to regulate CO via venous return.

### The functional view—a combination of anatomy and physiology

**Fig. 2:**
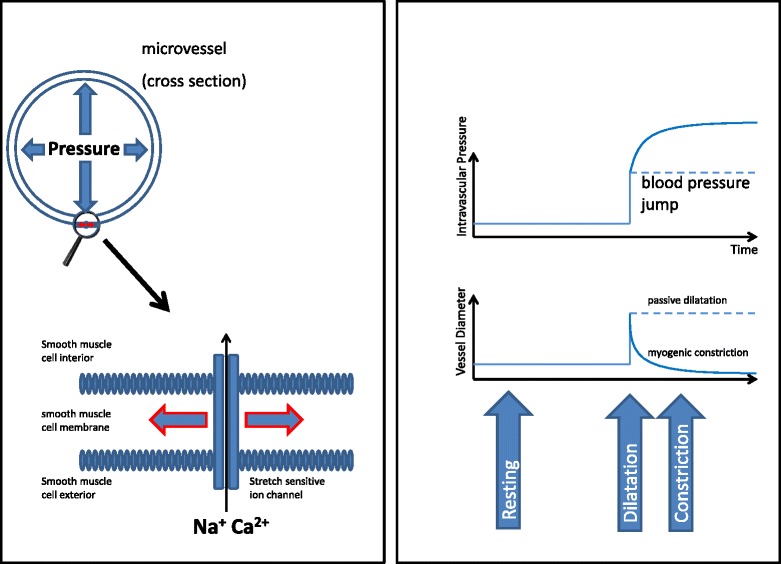
The myogenic response (Bayliss effect) as an example for vascular autoregulation. Dilatation of the microvessel leads to ion influx (Na^+^, Ca^2+^) through stretch-sensitive membrane ion channels and, therefore, to contraction of the vessel smooth muscle cells due to depolarisation (*left hand panel*, a very simple illustration of the reality where the link between stretch and smooth muscle contraction is certainly more complex). The *right hand panel* shows the impact of an acute increase in blood pressure on intravascular pressure and vessel diameter with (*full line*) and (potentially) without (*dotted line*) myogenic response. The Bayliss effect which targets maintaining tissue blood flow in the face of different blood pressure levels can be blocked, e.g., pharmacologically by calcium antagonists

**Fig. 3:**
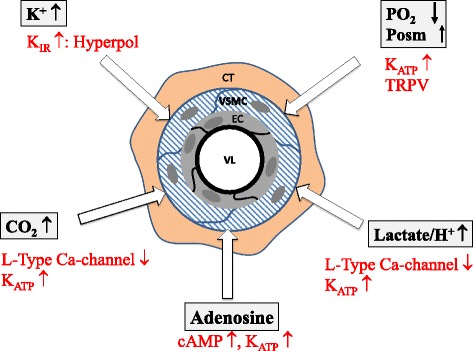
Local vasodilatation related to tissue metabolic activity. Local metabolic effects targeting a close relation of regional blood flow to metabolic activity are particularly effective in the terminal arterioles, being elicited foremost by changes in pO_2_, pCO_2_, pH, osmolarity, potassium ion concentration, and released catabolites such as adenosine. The respective signaling mechanisms are colored in *red. cAMP* cyclic adenosine monophosphate; *CT* connecting tissue, *EC* endothelial cell, *K*_*ATP*_ ATP-dependent potassium ion channel, *K*_*IR*_ inward-rectifying potassium ion channel, giving rise to hyperpolarization (Hyperpol), *Posm* osmotic pressure, *TRPV* transitory receptor-mediated potential, vallinoid type, *VL* vascular lumen, *VSMC* vascular smooth muscle cell

Four main characteristics can be attributed to the different microvascular sections: resistance, exchange, shunting ability, and capacitance. Traditionally, the smooth muscle tone of the precapillary resistance vessels has been attributed to mechanical autoregulation of blood flow countering changes in blood pressure. This resistance adaptation to systemic blood pressure was first impressively described by Bayliss [26]: an increase in blood pressure at the arteriolar level is directly linked to an increase in vascular smooth muscle tone in order to keep blood flow to chosen organs (foremost the brain, heart, kidney, liver, carotid bodies) constant over a wide pressure range, provided there is no change in organ function. A drop in blood pressure has the opposite effect. Figure 2 exemplifies this response, which is caused by alterations in ion transport (Na^+^, Ca^2+^) through stretch-sensitive membrane ion channels. As listed in Table 1, further effectors of arteriolar resistance are the autonomic nervous systems (generally adrenergic; cholinergic and non-adrenergic/non-cholinergic nerves being restricted to genital organs and the gastro-intestinal tract). Vasoactive humoral and tissue agents include angiotensin II, bradykinin, vasopressin, free catecholamines, natriuretic peptides, and many more, all acting via receptor-operated channels of VSMC and endothelial cells. Local metabolic effects, particularly effective in the terminal arterioles (Fig. 1), are elicited foremost by changes in pO_2_, pCO_2_, pH, osmolarity, potassium ion concentration, and released catabolites such as adenosine. Figure 3 illustrates their respective signaling cascades and modes of action. Finally, shear stress evoked by the movement of blood and impinging primarily at the endothelial surface causes the release of the dilatory autacoid nitric oxide (NO) [27]. This is a positive feedback mechanism: dilatation induced locally at terminal arterioles, e.g., by metabolic signals, increases flow, thus increasing shear stress and liberation of NO upstream. This NO acts both at the primary site of generation and downstream, furthering dilatation. Recent experimental evidence has shown that the endothelial glycocalyx is paramount in mediating mechanotransduction in this setting [28]. Accordingly, shedding of the glycocalyx as caused by inflammation, ischemiad and other pathological states will attenuate dilatation and local regulation of flow.

Numerous vasoactive substances elicit generation of the vasodilatatory autacoids NO and prostaglandin I_2_ (PGI_2_) via endothelial receptors found on endothelial cells of most sections of the vascular tree but foremost on those of terminal arterioles (Fig. 1). Known agonists include serotonin, histamine, adenine nucleotides ATP and ADP, bradykinin, acetylcholine, thrombin, and endothelin, along with many more. Interestingly, an intact endothelial lining hinders most of these substances from passing from the luminal into the interstitial space. For example, endothelial ectonucleotidases rapidly degrade adenine nucleotides to adenosine, a powerful vasodilator and inhibitor of platelet activation, as are NO and PGI_2_ [29]. Opposed to the endothelium-mediated vasodilatory actions, most of the aforementioned agonists elicit vasoconstriction when they gain direct access to the abluminal VSMC since the corresponding receptors on the smooth muscle cells activate calcium influx and the IP_3_-diacylglycerol pathways. A case in point is when endothelin, generated in endothelial cells following stimulation, e.g., by adrenaline, thrombin, or angiotensin II, is released into the subendothelial interstitial space [30]. It is then able to elicit its better-known, strong vasoconstrictive effect. A “leaky” vascular barrier will obviously change vascular responses for agonists arriving from the intraluminal side, allowing for more of a vasoconstrictive response. Another familiar vasoconstrictor is angiotensin II. This peptide can be formed directly in the interstitial space from precursor molecules by the enzymes chymase and angiotensin converting enzyme (ACE). However, ACE is also expressed as an ectoenzyme, especially on pulmonary endothelial cells. Thus, local generation of angiotensin II and cleavage of bradykinin by ACE at the vessel wall may also facilitate vasoconstriction indirectly [31].

To sum up the two previous paragraphs, it is important to note that many organs in the body differ with respect to the endowment of their vascular beds with membrane receptors and enzymes such as ACE. Thus, depending on the specific receptor expression (type and density) on endothelial as opposed to smooth muscle cells, the site of generation of the vasoactive substance (luminal or abluminal), the concentration of the agonist, and the leakiness of the vascular barrier, one and the same substance can elicit vasodilatation, vasoconstriction, or no net effect.

A totally different aspect is whether changing blood flow and flow distribution will ensure that the tissue in contact with the capillaries is actually making use of the offered blood flow for exchange. This is not guaranteed, especially when not under pathological situations, and present day clinical parameters may not be able to reflect this [32]. Experimental work on the animal brain revealed identical values of hemoglobin oxygen saturation in different venules draining the cortex, despite 20-fold differences in blood flow rate [33]. In a clinical study on septic shock patients, venous oxygen saturation and CO also seemed normal, while the p_v-a_CO_2_ showed an increase [15]. Interestingly, the increase in p_v-a_CO_2_ correlated well with parameters of microvascular disturbance, established by means of SDF imaging of sublingual microvessels [34]. Ospina-Tascon et al. have shown that the persistence of a high p_v-a_CO_2_ (≥6 mmHg) during the first 6 h of resuscitation of septic shock patients was associated with higher incidences of multiple organ failure and mortality [35].

Thus, what we can see with pulse oximetry or when measuring central venous oxygen saturation (ScvO_2_) is, first of all, a “generalized” global situation. Should this appear normal, although one must suspect that the tissues need to extract more of the available oxygen than under steady-state conditions, we cannot take such measures at face value. Phenomena such as systemic microvascular shunting may falsify the global result. Small organs, such as the heart or the kidneys especially, can already be severely in trouble despite a ScvO_2_ greater than 75 %. There is consensus about evaluating the state of the microvasculature, based on parameters such as microvascular flow index, heterogeneity index, total vascular density, and functional capillary density [36]. However, the technique of SDF implemented to perform such measurements is not generally available at the bedside and, moreover, has not been clinically verified in larger studies. The greatest uncertainty associated with SDF as it is performed today probably exists with respect to the extrapolation of measurements conducted on sublingual or nailfold vessels to the body in general and, especially, to organs at risk.

### Diffusion and filtration—the two principles of microvascular exchange, the final goal of perfusion

Rates of mass transfer between compartments or phases depend linearly on the exchange area. Additionally, diffusion coefficients, membrane carrier or channel-mediated transport, and barrier permeability plus solvent drag can come to bear in specific cases. Diffusion is the quantitatively most important form of capillary exchange activity for small, lipid-soluble molecules, especially the respiratory gases, since their diffusional mobility is extremely high and the distance that needs to be overcome is small, e.g., about 1–2 μm between erythrocyte and endothelial cell basement membrane. This normally allows for a complete equilibration between blood and the surrounding interstitial space during the passage through the microvasculature. In the lung, for instance, the respiratory gases have already equilibrated between the alveolar space and the blood after about one-third of the pulmonary passage time available to blood at resting CO. Without capillary recruitment, however, i.e., a pronounced enlargement of exchange area, equilibration at 4–5-fold heightened CO would no longer occur. In the special case of water, transport via aquaporins can be the quantitatively significant mode of exchange in microvascular segments with extremely tight endothelium, as in the brain [37]. Most other organs and tissues have more leaky microvessels, so that paracellular filtration of water plays the major role in transport. High hydraulic conductivity is found especially in glomerular capillaries [38] and in the liver, but practically no vascular bed is completely impermeable to water. One can be fatalistic about this and say that it just was not worth it for nature to go to the trouble of making vessels impermeable. However, exchange of fluid across the vascular wall—in both directions—is an enormously vital physiological function.

### Fluid homeostasis

**Fig. 4:**
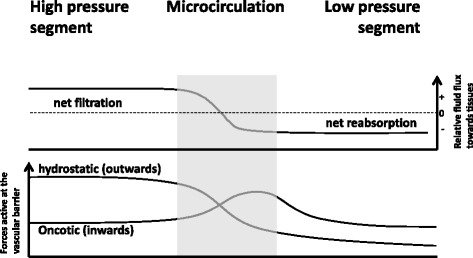
The principle of Ernest Starling. The high filtration-high reabsorption scenario proposed by Ernest Starling presumed high filtration in the high-pressure segments due to outweighing hydrostatic forces and reabsorption of a very large part of the filtered volume at the venular aspect owing to prevailing oncotic forces in the lumen. Fluid excess in the interstitial space needs to be drained by the lymphatic system (according to Becker et al. [28])

According to Ernest Starling, filtration occurs mainly at the arteriolar side of the capillaries, a large part of the fluid being reabsorbed at the venular aspect so as to avoid tissue edema (Fig. 4) with the balance of fluid in the interstitial space being transported back into the large venous circulation via the lymphatic system [39]. Driving forces in this classical concept are the intravascular hydrostatic pressures (higher in the arteriolar segment than at the venular end), the interstitial hydrostatic pressure, and the opposing oncotic pressures (high intravascular and, by definition, low interstitial). The oncotic pressure, the force drawing water across a semi-permeable barrier, is really a combination of two effects: the presence of macromolecules such as proteins, nucleic acids, polyphosphates, and polysulfated moieties, all with relatively low permeability across the vascular wall, and the Gibbs-Donnan equilibrium established by charged constituents such as albumin (page 23 in [19]). These attract smaller counter ions in alternatingly charged spheres and clouds, thereby increasing the total osmotic effect on water beyond that expected simply on the basis of macromolecule number (page 436 in [19]).

**Fig. 5:**
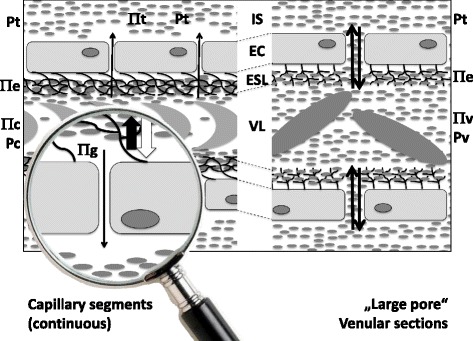
The endothelial surface layer model. *Left hand panel:* An intact endothelial surface layer, consisting of the endothelial glycocalyx and attached plasma protein molecules, oncotically (*thick black arrow*) limits hydrostatically driven (*thick white arrow*) fluid movement across the vascular wall within the microvascular high-pressure segments, which, in addition to narrow interendothelial clefts with high resistance to water flow, allows for hardly any egress of colloidal particles and only very low net rates of fluid extravasation (*thin black arrow*; Πt and Πc are in equal ranges, but irrelevant because Πe (high) and Πg (low) count). *Right hand panel:* At the venular aspect, relatively free and easy exchange of colloidal particles is allowed in both directions across the vascular wall (*black arrows*). This is feasible, because the interstitial space of most organs and tissues is now known to possess oncotic and hydrostatic pressures close to those existing in the end- and post-capillary vessel segments (Πv – Πt is small, but Pv – Pt is also small). There is no need for largescale reabsorption, as suggested by Ernest Starling (according to Jacob et al. [41] and Becker et al. [28]). *EC* endothelial cell, *ESL* endothelial surface layer, *IS* interstitial space, *Πc, e, g, t, and v* oncotic pressure in capillary plasma, ESL, below the ESL, in the tissue, and venular space, respectively, *Pc, t, and v* hydrostatic pressure in the capillary, tissue, and venule, respectively

Meanwhile, interesting new models integrating an endothelial glycocalyx tightening the system mainly at the arteriolar site and clothing large pores in the low-pressure segments have emerged, replacing the classical high filtration-high reabsorption scenario promoted by Starling (Fig. 5) [28, 40, 41]. Table 2 lists a number of physiological and pathophysiological functions and involvements of the glycocalyx. This, at first sight an anatomically somewhat insignificant structure, also provides an answer to the puzzling finding made several years ago that the interstitial oncotic pressure appears to be close to that of the intravascular region. Because of its relatively low permeability to plasma proteins, the glycocalyx creates a zone of low oncotic pressure directly at the endothelial surface. It is thus the oncotic gradient across the glycocalyx from the plasma space to the cell membrane surface that generates the real opposition to the hydrostatic filtration forces acting from the intra- to the extravascular space [40–42].

**Table 2:** The endothelial surface layer

A) Functions • Regulation of vascular permeability • Mediation of shear stress • Attenuation of leukocyte and platelet adhesion • Binding of cytokines/chemokines, hormones, etc.	
B) Scenarios of damage • Ischemia/reperfusion, hypoxia, shock • Inflammation • Volume loading • Chronic heart failure (due to release of natriuretic peptides) • Atherosclerosis and diabetes • Radiation (?)	
C) Present and potential future strategies of clinical protection • Plasma albumin in organ transplantation • Hydrocortisone and mast cell inhibition • Direct cytokine inhibition • Antithrombin and protease inhibitors • Avoidance strategies (antioxidants, normovolemia) • Supply of “prefabs” (e.g., heparinoids, Danaparoid) • Anesthetic regimens (e.g., sevoflurane)	

Adapted from Becker et al. [28]

Nevertheless, it still appears valid that the main driving force of filtration is the hydrostatic pressure within the early capillary section, having been measured as 30–35 mmHg at the beginning, and decreasing to 13–17 mmHg close to the venular end. The opposing oncotic pressure resulting from a normal plasma protein concentration of about 70 g/l (which includes about 4 g/l of albumin) is around 25 mmHg [19]. This leads to a slight net overweight of the outwardly directed force at the start of the capillaries. However, very narrow interendothelial clefts, furthermore ribbed by membrane strands with only small gaps, additionally impede fluid flux. Still, the outward flow through the cleft is important, because it prevents movement of colloids from the interstitial space up towards the base of the apical glycocalyx. Such movement would otherwise eliminate the oncotic gradient established across the glycocalyx. In the venular section, there is also the gradient of oncotic pressure between the luminal and basal aspect of the glycocalyx. This favors flow of fluid from the interstitial to the intravascular space. However, owing to the larger interendothelial clefts present in the venular beds of most organs and the relatively high interstitial oncotic pressure now known to exist in most organs, little driving force for fluid filtration remains (see Fig. 5, right hand panel). The physiologically observed resultant small net fluid loss from the microvascular exchange section can be efficiently drained back towards the circulation as long as the lymphatic system works properly. Under normal circumstances, the resistance of the interstitial space against being passively loaded with fluid is high, together with a high capacity of the lymphatic system to increase fluid removal in response to an increasing interstitial pressure. This explains why an increasing net outwards directed filtration force does often not lead directly to interstitial edema [28].

The role of venous resistance in the regulation of fluid extravasation is uncertain. First of all, one must concede that in the systemic circulation there is certainly nothing akin to the situation found in the kidneys for regulating glomerular filtration. There we have artery-typical vessel structures in both pre- and post-glomerular arterioles. In the systemic circulation, on the other hand, the histological profile of pre- and post-capillary vessels is different. Though sympathetic nervous innervation occurs in both the arterial and the venous system, the strength of induced vasoconstriction is much greater in the arterial segment, largely owing to the density of innervation and the far richer endowment of arterial vessels with smooth muscle cells. Other than that, nothing much is known about possible reactions of the venules and small collecting veins to vasoactive stimuli. Homeostasis of fluid exchange in the microvasculature alone on the basis of actively balanced reactions of venular to arteriolar blood pressure does not seem to be a principle used physiologically by man. With respect to scenarios of circulatory shock, however, certain alterations in the microvascuature have been experimentally verified. Especially during the first phase of hemorrhage, a sypathetic nervous reaction reduces microvascular perfusion in non-essential organs and, thereby, initially reduces fluid extravasation. This outweighs effects from the venous side, the vessels of which also constrict slightly. With ongoing development of shock, however, tissue hypoxia increases general vascular permeability, enhancing leak into the interstitial space even at low intravascular hydrostatic pressure. The proven ability of small-volume resuscitation with hyperoncotic albumin to return volume to the intravascular space shows that permeability effects are present [43].

Hemodynamic consequences of tissue edema merit further discussion here. Tissue edema will be expected to elevate microvascular resistance to flow because the interstitial pressure rises. Provided precapillary resistance vessels are still able to regulate, precapillary intravascular hydrostatic pressure may be expected to rise to compensate, keeping blood flow and fluid extravasation at a similar level as without edema. On the other hand, postcapillary intravascular hydrostatic pressure probably will not increase as long as venular outflow is unimpeded. According to both the old (Starling) and new concept (glycocalyx) of microvascular fluid homeostasis, this then amounts to an enhancement of fluid egress from the interstitial space into the venular segments and, thus, to some attenuation of fluid accumulation in the edematous tissue. Intuitively, early hemorrhagic shock and septic shock should be expected to differ. In the former, reactive arterioconstriction will reduce precapillary hydrostatic pressure, thus reducing fluid filtration into the interstitial space. In the latter, systemic vasodilatation will enhance precapillary pressure. On top of that, shedding of the glycocalyx leads to breakdown of the oncotic pressure gradient at the endothelial surface, and inflammatory mediators generally induce widening of the interendothelial clefts. They also directly and indirectly enhance sticking of inflammatory leukocytes and of blood platelets to the walls of venules and collecting veins, thereby effectively leading to a narrowing of the microvascular outflow tract [22, 28, 29]. All of this is bad news for fluid homeostasis. If there is a choice, then better to choose hemorrhagic rather than septic shock for yourself.

Clearly, damage to the endothelial glycocalyx should directly elevate microvascular hydraulic conductivity and enhance permeability towards all types of plasma constituents. Such damage occurs in situations of inflammation, hypoxia, postischemic reperfusion, volume expansion, and also mechanical manipulation of the heart, just to name the most common causes [44–47]. Natriuretic peptides have been linked to volume extravasation and have also been found to shed the glycocalyx in coronary bypass surgery [44, 45]. Recently, studies linking glycocalyx damage and the functional state of the microvasculature have been forthcoming in studies conducted in man [48]. It is not excessive to consider that the scientific community is at the beginning of a new age of microvascular understanding.

## Conclusions

This work endeavors to give an overview of our current knowledge about oxygen supply (DO_2_) to the tissues and about microvascular fluid exchange. The main message for the clinician has to be that feeling safe solely on the basis of having secured the airways and assessing and optimizing macrohemodynamics might be deceptive. Regrettably, it is often still the only option daily practice offers to us. Clinically, it is our turn to convince colleagues, hospitals, and sponsors that this is insufficient and that it is high time to give more attention to bedside techniques providing insight into local tissue perfusion. Scientifically, it is time to optimize the already available techniques for visualizing microcirculation at the bedside and to define representative tissues and normal values we can rely on, in order to use them for therapeutic decisions in the near future.

It is our hope and belief that only widespread insight into microvascular physiology and pathophysiology has the power to improve diagnostics, leading to a real target-oriented therapy of oxygen delivery in our critically ill patients.

## Abbreviations

ACE: Angiotensin converting enzyme
ADP: Adenosine diphosphate
ATP: Adenosine triphosphate
AV: Arteriovenous
CaO_2_: Arterial oxygen content
CHb: Hemoglobin concentration in the blood
CO: Cardiac output
CO_2_: Carbon dioxide
DO_2_: Oxygen delivery
FiO_2_: Fractional oxygen content of inspiratory gas
H^+^: Proton
H_2_O: Water
ICU: Intensive care unit
NAD^+^/NADH: Nicotinamide adenine dinucleotide oxidized/reduced
NO: Nitric oxide
O_2_: Oxygen
P: Phosphate
paCO_2_: Arterial partial pressure of carbon dioxide
p_amb_: Ambient air pressure
pAO_2_: Alveolar partial pressure of oxygen
PGI_2_: Prostaglandin I_2_
pH_2_O_sat_: Saturated water vapor pressure
pO_2_: Partial pressure of oxygen
p_v-a_CO_2_: Venous-to-arterial difference in the partial pressure of carbon dioxide
RQ: Respiratory quotient
SaO_2_: Arterial oxygen saturation
ScvO_2_: Central venous oxygen saturation
SDF: Sidestream Dark Field Imaging
VSMC: Vascular smooth muscle cells
